# Progesterone receptor isoforms A and B in human epithelial ovarian carcinoma: immunohistochemical and RT-PCR studies

**DOI:** 10.1054/bjoc.2000.1463

**Published:** 2000-11-22

**Authors:** J Akahira, T Inoue, T Suzuki, K Ito, R Konno, S Sato, T Moriya, K Okamura, A Yajima, H Sasano

**Affiliations:** Departments of Pathology, Obstetrics and Gynecology, Tohoku University School of Medicine, Sendai, Japan

**Keywords:** progesterone receptor, PRA, PRB, ovarian cancer, RT-PCR, immunohistochemistry

## Abstract

Human epithelial ovarian carcinoma is well-known as a sex steroid-dependent neoplasm, but the possible biological significance of progesterone receptor (PR) in this cancer remains controversial. Recently, two isoforms of human PR, PRA and PRB, have been characterized and different functional characteristics have been reported for these two isoforms. We therefore examined immunohistochemistry (107 cases) and reverse transcription-polymerase chain reaction (RT-PCR) (16 cases) for PRA, PRB, and oestrogen receptor-a (ER-a). Labeling indices (LI) for PRA and PRB were 2.4 and 43.6, respectively, and the difference was statistically significant. PRB LI, but not PRA LI, as well as performance status, stage, and residual tumour turned out to be independent prognostic factors following multivariate analysis. There was also a significant correlation between ER-a LI and PRB LI (r = 0.595, P < 0.0001), suggestive of a possible interaction between these two receptors. RT-PCR also detected the expression of PR isoform transcripts in the same pattern as was observed with immunohistochemistry. Results of these studies indicate that PRA and PRB both mediate distinct pathways of progesterone action in ovarian carcinoma. Moreover, it is important to examine PRB LI as a prognostic factor in the cases of human epithelial ovarian carcinoma. © 2000 Cancer Research Campaign http://www.bjcancer.com


*British Journal of Cancer* (2000) **83**(11), 1488–1494
© 2000 Cancer Research Campaign
doi: 10.1054/ bjoc.2000.1463, available online at http://www.idealibrary.com on IDEAL®
http://www.bjcancer.com

### Progesterone receptor isoforms A and B in human epithelial ovarian carcinoma: immunohistochemical and RT-PCR studies

**J Akahira[1,2], T Inoue[1], T Suzuki[1], K Ito[2], R Konno[2], S Sato[2], T Moriya[1], K Okamura[2], A Yajima[2] and H Sasano[1]**

Departments of [1]Pathology; and [2]Obstetrics and Gynecology, Tohoku University School of Medicine, Sendai, Japan

Summary Human epithelial ovarian carcinoma is well-known as a sex steroid-dependent neoplasm, but the possible biological significance of progesterone receptor (PR) in this cancer remains controversial. Recently, two isoforms of human PR, PRA and PRB, have been characterized and different functional characteristics have been reported for these two isoforms. We therefore examined immunohistochemistry (107 cases) and reverse transcription-polymerase chain reaction (RT-PCR) (16 cases) for PRA, PRB, and oestrogen receptor-a (ER-a). Labeling indices (LI) for PRA and PRB were 2.4 and 43.6, respectively, and the difference was statistically significant. PRB LI, but not PRA LI, as well as performance status, stage, and residual tumour turned out to be independent prognostic factors following multivariate analysis. There was also a significant correlation between ER-a LI and PRB LI ($r = 0.595$, $P < 0.0001$), suggestive of a possible interaction between these two receptors. RT-PCR also detected the expression of PR isoform transcripts in the same pattern as was observed with immunohistochemistry. Results of these studies indicate that PRA and PRB both mediate distinct pathways of progesterone action in ovarian carcinoma. Moreover, it is important to examine PRB LI as a prognostic factor in the cases of human epithelial ovarian carcinoma. © 2000 Cancer Research Campaign http://www.bjcancer.com

**Keywords**: progesterone receptor; PRA; PRB; ovarian cancer; RT-PCR; immunohistochemistry

Epithelial ovarian carcinoma is the leading cause of death from gynaecological malignancies in the great majority of developed countries (Nakashima et al, 1990). This high mortality is considered to be, in large part, due to the advanced stage of the disease commonly present at the time of diagnosis, but many clinical studies have reported that there are some prognostic factors in ovarian carcinoma other than clinical stages, such as histology, the degree of primary surgical cytoreduction, response to chemotherapy, and others (Young et al, 1978; Redman et al, 1986; Heintz et al, 1986; Piver et al, 1988; Omura et al, 1991; Del Campo et al, 1994).

Sex steroid hormones have been implicated in the aetiology and/or progression of some epithelial ovarian cancers. Both progesterone (PR) and oestrogen receptors (ER) have been reported in human epithelial ovarian carcinoma (Rao and Slotman, 1991). In endometrioid endometrial and breast carcinoma, steroid hormone receptor status correlates well with response to hormonal manipulation and prognosis (McGuire et al, 1978; Benraad et al, 1980; Bloom et al, 1980; Osborne et al, 1980; Ehrlich et al, 1981; Kaupilla, 1984). However, in epithelial ovarian carcinoma, the prognostic significance of tumour ER and PR status among patients still remains controversial (Bizzi et al, 1988; Masood et al, 1989; Sevelda et al, 1990; Rao and Slotman, 1991; Hempling et al, 1998).

PR is a member of a subgroup of nuclear receptors which regulate a number of physiological and morphological processes in response to binding of their ligands (Evans, 1988). There are two isoforms of the human PR, PRA and PRB, which differ only in that the smaller isoform, PRA, lacks the N-terminal 164 amino acids of the larger isoform, PR-B (Horwitz and Alexander, 1983; Jeltsch et al, 1986; Savouret et al, 1990; Kastner et al, 1990). PRA and PRB are products of a single gene and are translated from an individual messenger RNA species under the control of distinct promoters (Kastner et al, 1990). The transcription of both isoforms is indirectly induced by oestradiol via ER (Kastner et al, 1990). Both PRA and PRB function as ligand-activated transcription factors but several in vitro studies have demonstrated different functional characteristics between these two isoforms. For instance, transcriptional activation of the progesterone responsive element-containing promoters by PRB is more marked than that by PRA, although the differences are cell-specific (Kastner et al, 1990; Wen et al, 1994; Giangrande et al, 1997). In addition, PRA can also act as a transcriptional inhibitor of other steroid hormone receptors, including ER and PRB (Wen et al, 1994; Giangrande et al, 1997; Vegeto et al, 1993; Kraus et al, 1995). Results of these studies indicate that the relative levels of PRA and PRB within target cells may determine the nature and functional responses to progesterone.

In the normal human endometrium (Mote et al, 1999; Critchley et al, 1998; Mangal et al, 1997; Wang et al, 1998), leiomyoma (Viville et al, 1997; Fujimoto et al, 1998) and endometrial carcinoma (Kumar et al, 1998; Fujimoto et al, 1995) several studies have examined the relative abundance of PRA and PRB expression, and all studies to date support the presence of distinct pathways of progesterone actions in these tissues. However, the expression of PRA and PRB in epithelial ovarian cancer and their clinical significance have not been examined. Therefore, in this

*Received 27 March 2000*
*Revised 17 July 2000*
*Accepted 18 July 2000*

*Correspondence to*: J Akahira

**Table 1** Correlations between clinical characteristics and hormone receptor immunoreactivity

| | *n* | Median labelling indices (range) | | | |
|---|---|---|---|---|---|
| | | PRAB | PRA | PRB | ER[b] |
| Total[a] | 107 | 42.4 (0–100) | 2.4 (0–58.4) | 43.6 (0–93.2) | 12.8 (0–85.2) |
| Age | | | | | |
| < 50 years | 47 | 44 (0–100) | 4 (0–58.4) | 47.2 (0–92) | 12.8 (0–85.2) |
| ≥ 50 years | 60 | 41.6 (0–96) | 2.2 (0–48) | 40.2 (0–93.2) | 12.4 (0–63.6) |
| Performance status | | | | | |
| 0–1 | 76 | 47.6 (0–100) | 2.7 (0–58.4) | 45.6 (0–93.2) | 16.2 (0–85.2) |
| 2–4 | 31 | 30 (0–90) | 0 (0–35.2) | 25.2 (0–90) | 4 (0–63.6) |
| Histology | | | | | |
| Serous | 46 | 52.8 (0–100) | 2.4 (0–43.2) | 51.2 (0–93.2) | 22.4 (0–85.2) |
| Endometrioid | 18 | 40 (0–82) | 0 (0–52) | 39.2 (0–75.6) | 0 (0–49.6) |
| Clear cell | 25 | 32.4 (0–94) | 0 (0–35.2) | 31.2 (0–92) | 8.8 (0–54) |
| Mucinous | 18 | 55.8 (0–100) | 7.4 (0–58.4) | 39.7 (0–82) | 23.4 (0–67.2) |
| Stage | | | | | |
| I–II | 50 | 47.4 (0–100) | 3 (0–58.4) | 43.2 (0–92) | 16.2 (0–67.2) |
| III–IV | 57 | 34 (0–100) | 2.4 (0–52) | 43.6 (0–93.2) | 10.4 (0–85.2) |
| Grade | | | | | |
| 1 | 44 | 51.2 (0–100) | 2.6 (0–52) | 43.6 (0–92) | 8.8 (0–68) |
| 2 | 40 | 42.4 (0–100) | 2.4 (0–58.4) | 43.6 (0–90) | 14.2 (0–54) |
| 3 | 23 | 39.8 (0–96) | 0 (0–35.2) | 38.8 (0–93.2) | 14 (0–85.2) |
| Residual tumour | | | | | |
| Optimal | 77 | 48 (0–100) | 4 (0–58.4) | 45.6 (0–92) | 14.8 (0–85.2) |
| Suboptimal | 29 | 27.4 (0–96) | 0 (0–31.2) | 24 (0–93.2) | 4.4 (0–59.2) |

[a]Difference of total labelling indices (LI) between PRA and PRB is significant ($P < 0.01$); [b]Significant difference of LIs was observed for performance status and histology ($P < 0.05$); Performance status score: 0 = asymptomatic and fully active; 1 = symptomatic, fully ambulatory, restricted in physically strenuous activity; 2 = symptomatic, ambulatory, capable of self-care, more than 50% of waking hours are spent out of bed; 3 = symptomatic, limited self-care, spends more than 50% of time in bed, but not bedridden; 4 = completely disabled, no self-care, bedridden study, we evaluated the expression of PRA and PRB in human epithelial ovarian carcinoma using immunohistochemistry and RT-PCR, and their significance as prognostic indicators in epithelial ovarian cancer. We also evaluated the relationship between PR isoforms and ER-α immunohistochemically, because of the unique in vitro activation–inhibition interactions of these sex steroid receptors described above.

### MATERIALS AND METHODS

We studied a total of 107 cases of common epithelial ovarian carcinoma. The clinicopathological features of the patients examined are summarized in Table 1. Information regarding age, performance status on admission, histology, stage, grade, residual tumour after primary surgery, response to primary chemotherapy, and overall survival was retrieved from the review of patient charts. Median follow-up time of the patients in this study was 54 months (18–112 months). Seventy-seven patients (71.9%) were optimally cytoreduced at the time of surgery. The great majority of patients (83.2%) received platinum-containing chemotherapy, and approximately half of them (49.4%) received chemotherapy consisting of cisplatin, adriamycin and cyclophosphamide (CAP). Moreover, more than half of the patients (62.5%) who had measurable disease after primary surgery responded to chemotherapy. It was difficult to evaluate the response to primary chemotherapy in relation to survival because 75 of the 107 patients (72.0%) were optimally cytoreduced with primary surgery, and thus they could not be evaluated (NE) on their response to chemotherapy. Performance status was defined according to WHO criteria (World Health Organization, 1979). Histology, stage and grade were determined according to FIGO criteria (International Federation of Gynecology and Obstetrics, 1987; Pettersson, 1994). Residual disease was determined by the amount of unresectable tumour left following primary cytoreductive surgery. Optimal cytoreduction was defined as no gross residual tumour greater than 2 cm in diameter, whereas suboptimal cytoreduction was defined as any gross residual disease remaining greater than 2 cm in diameter. Response to primary chemotherapy was assessed according to WHO criteria (World Health Organization, 1979). Overall survival was calculated from the time of initial surgery to death, or the date of last contact. Survival times of patients still alive or lost to follow up were censored in February 2000. All of these archival specimens were retrieved from the surgical pathology files at Tohoku University Hospital, Sendai, and Miyagi Prefectural Cancer Center, Natori, Japan. These specimens were all fixed in 10% formalin and embedded in paraffin. Among these 107 cases, 16 cases were available for examination by reverse transcription-polymerase chain reaction (RT-PCR) analysis. These specimens were dissected immediately into small pieces following gross dissection, quickly transferred to liquid nitrogen, and then stored at –80°C until further use. The research protocol was approved by the ethics committee of Tohoku University School of Medicine, Sendai, Japan.

### Immunohistochemistry

Immunohistochemical analysis was performed using the streptavidin–biotin amplification method using a Histofine Kit (Nichirei, Tokyo, Japan), and have been previously described in detail (Hirasawa et al, 1997). The characteristics of the primary antibodies employed in this study are summarized in Table 2. The hPRa2 and hPRa3 antibodies recognize PRB and PRA and B, respectively. The hPRa7 antibody employed in this study recognizes both PRA and PRB on immunoblot analysis (Clarke et al,

© 2000 Cancer Research Campaign 

**Table 2** Primary antibodies employed in immunohistochemistry

| Antibody | Source | Optimal dilution | Antibody retrieval |
|---|---|---|---|
| PRA and B: hPRa3 (Monoclonal) | Neomarkers (California, USA) | 1:50 | Autoclave[a] |
| PR A: hPRa7 (Monoclonal) | Neomarkers (California, USA) | 1:100 | Autoclave[a] |
| PRB: hPRa2 (Monoclonal) | Neomarkers (California, USA) | 1:100 | Autoclave[a] |
| ER α (Monoclonal) | Immunotech (Marseille, France | 1:5 | Autoclave[a] |

[a]Heat in an autoclave for 5 min in citric acid buffer (2 mM citric acid and 9 mM trisodium citrate dehydrate, pH 6.0)

1987), but specifically recognizes PRA when utilized for immunohistochemistry (Mote et al, 1999). The ER antibody recognizes ER-α, the traditinal estrogen receptor, but not ER-β. The antigen–antibody complex was visualized with 3,3′-diaminobenzidine (DAB) solution (1 mM DAB, 50 mM Tris-HCl buffer, pH 7.6, and 0.006% $H_2O_2$), and counterstained with haematoxylin. Proliferative-phase endometrial glands were used as positive controls for PR isoforms and ER-α (Clarke et al, 1987). As negative controls, 0.01 M phosphate buffered saline and normal mouse IgG were used in place of primary antibodies. No specific immunoreactivity was detected in these tissue sections.

### Scoring of immunostaining

For evaluation of PRA, PRB, PRAB and ER-α immunoreactivity, labeling index (LI) was obtained in carcinoma cells as described by Sasano et al (1996). We obtained LI in the following manner. Two of the authors (JA and TM) independently evaluated at least 500 carcinoma cells microscopically. These fields of evaluation were determined by these two authors prior to evaluation using double-headed light microscopy. The mean value was obtained when interobserver differences were less than 5%. Immunostained slides were simultaneously evaluated using double-headed light microscopy when interobserver differences were greater than 5%. Intraobserver differences were less than 5% in this study.

### RT-PCR

Total RNA was extracted by homogenizing tissue specimens in guanidinium thiocyanate followed by ultracentrifugation in caesium chloride, as described previously (Sambrook et al, 7.1–7.87), and quantified spectrophotometrically at 260 nm. An RT-PCR kit (SUPERSCRIPT Preamplification system, Gibco-BRL, Grand Island NY, USA) was employed in the synthesis and amplification of cDNA. cDNAs were synthesized from 5 µg of total RNA using oligo (dT) primer and reverse transcription was carried out for 50 min at 42°C with SUPERSCRIPT II reverse transcriptase. After an initial 1 min denaturation step at 94°C, 35-cycle PCRs were carried out on a DNA thermal cycler (PTC-200 DNA Engine, MJ Research Inc, USA) under the following conditions: 1 min denaturation at 94°C, 1 min annealing at 56°C, and a 2 min extension at 72°C. Primers for PCR reactions were as follows; PRB (Kumar et al, 1998): 5′ sense-ACAGAATTCATGACTGAGCTGAAGGCAAAGGGT and 3′ antisense-ACAAGATCTCAAACAGGCACCAAGAGCTGCTGA (744–1173, 429 bp); PRAB (Kumar et al, 1998): 5′ sense-ACAGAATTCA TGAGCCGGTCCGGGTGCAAG and 3′ antisense-ACAAGATCTCCACCCAGAGCCCGAGGT TT (1239–1482, 243 bp); β-actin (Willey et al, 1998): 5′ sense-GATTCCTATGTGGGCGACGAG and 3′ antisense-CCATCTCTTGCTCGAAGTCC (192–723, 532 bp). β-actin primers were utilized as positive controls. Negative controls without RNA and without reverse transcriptase were also performed.

### Statistical analysis

Statistical analysis was performed using Stat View 5.0 (SAS Institute Inc, North Carolina, USA) software. The statistical significance of association between hormone-receptor status and characteristics of the patients was evaluated using a Mann–Whitney U-test, Kruskal–Wallis, and Scheffe analysis. Correlation among scored PR isoforms and ER-α immunohistochemistry was also assessed using a Spearman rank correlation. Univariate analysis of prognostic significance for prognostic factors was performed using a log-rank test, after each survival curve was obtained by the Kaplan–Meier method. Multivariate analysis of survival time was performed with Cox's proportional hazards model. All patients who could be assessed were included in the intention-to-treat analysis. A result was considered significant when the *P* value was less than 0.05.

### RESULTS

Results of immunohistochemistry are summarized in Table 1. Immunoreactivity for PR isoforms and ER-α were confined exclusively to the nuclei of tumour cells. No immunoreactivity, however, was detected in stromal cells (Figure 1). Median LI for PRB was 43.2% (range 0–93.2%), while that of PRA was 2.4% (0–58.4%). In all histological types and age groups, PRB LI was significantly higher than PRA LI ($P < 0.01$). No significant correlations were detected between PRAB, PRA or PRB LI and any of the clinicopathological parameters in the patients examined in this study. There was a highly significant correlation between ER-α and PRB LI ($r_s = 0.60$, $P < 0.0001$), and a weak correlation was also detected between ER-α and PRA LI ($r_s = 0.28$, $P = 0.038$, Figure 2).

Results of univariate analysis of prognostic significance for each variable, with respect to survival, are summarized in Table 3. In this analysis, we determined the positive cases as those with an LI of more than 10%. There were 35.5% and 71.9% PRA- and PRB-positive cases, respectively. Among the clinicopathological factors examined, those significantly associated with overall survival were histology, stage, performance status, residual tumour, PRAB, PRB, and ER-α immunoreactivity. In multivariate

 © 2000 Cancer Research Campaign

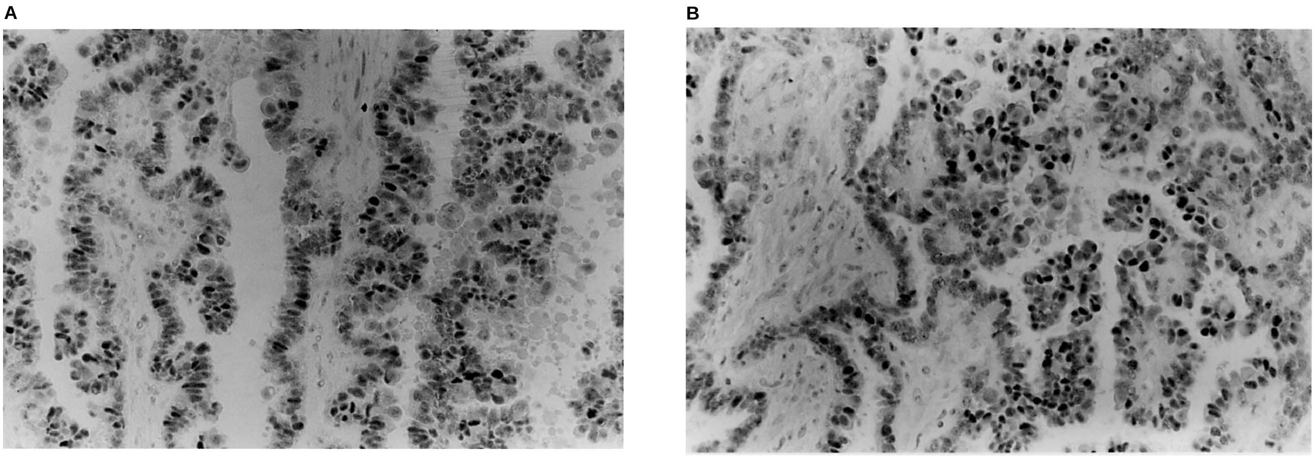

**Figure 1** Immunohistochemistry for (**A**) PRAB and (**B**) PRB in ovarian serous adenocarcinoma obtained from a 47-year-old patient, stage IIIc. Marked nuclear immunoreactivity was detected for PRAB (**A**) and PRB (**B**) in this case (magnification × 200)

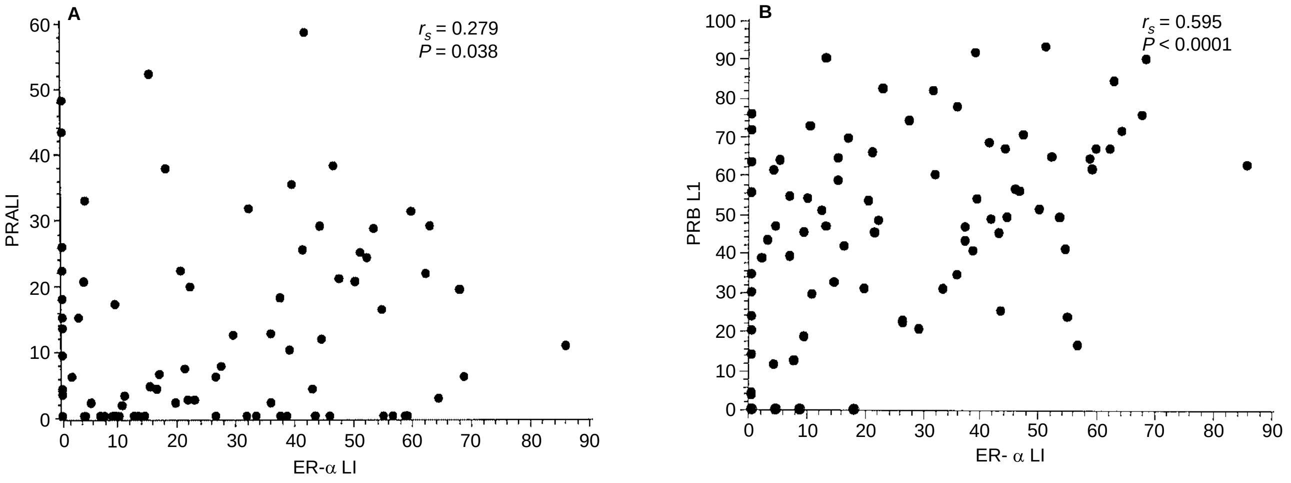

**Figure 2** Correlation between (**A**) PRA and (**B**) PRB labeling index (LI) and ERα LI in ovarian cancers. Both PR isoforms and ER-α LI were compared by the Spearman rank correlation test. Several points in the Figures (**A** and **B**) overlap each other because of the same values

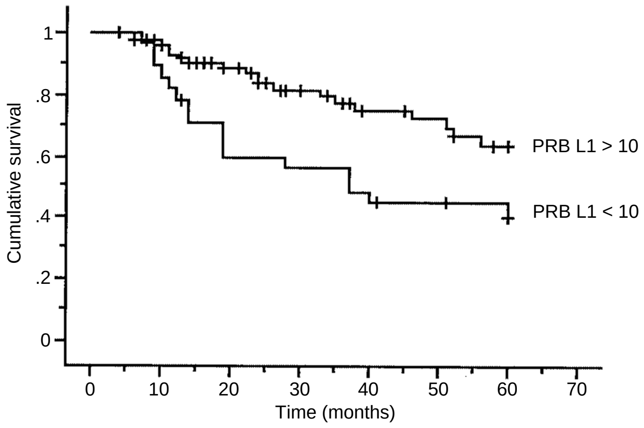

**Figure 3** Correlation between PRB labelling index (LI) and survival of patients with epithelial ovarian carcinoma. PRB LI was determined as described in the 'Materials and Methods'. Kaplan–Meier curves were compared

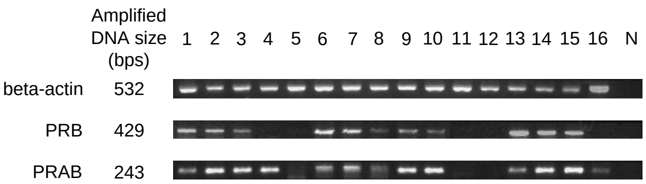

**Figure 4** RT-PCR analysis of total RNA extracted from ovarian epithelial carcinoma cases. Bands of the correct size for PRAB (243 bp) and PRB (429 bp) were detected in each histological subtype of ovarian epithelial carcinoma (Lanes 1–5 = serous: lanes 6–8 = mucinous; lanes 9–11 = endometrioid; lanes 12–16 = clear cell). Positive (β-actin) and negative (N) controls are also shown.

analysis, PRB immunoreactivity was a significant ($P$ = 0.037) predictor of overall survival but not PRAB or ER-α (Table 4, Figure 3). Performance status, stage, and residual tumour all turned out to be independent prognostic factors. Results of RT-PCR analysis for each histologic subtype are summarized in Figure 4. The expression of the mRNAs for PRAB and PRB are: serous, 4/5 and 3/5; mucinous, 3/3 and 3/3; endometrioid, 2/3 and

© 2000 Cancer Research Campaign

**Table 3** Univariate analysis of overall survival

| Factor | *n* | Median survival (months) | *P* |
|---|---|---|---|
| Age | | | |
| < 50 years | 47 | > 60 | 0.9 |
| ≥ 50 years | 60 | 56 | |
| Performance status | | | |
| 0–1 | 76 | > 60 | < 0.0001 |
| 2–4 | 31 | 22 | |
| Histology | | | |
| Serous | 46 | 60 | 0.0375[a] |
| Endometrioid | 18 | 37 | |
| Mucinous | 18 | > 60 | |
| Clear cell | 25 | > 60 | |
| Stage | | | |
| I–II | 50 | > 60 | < 0.0001 |
| III–IV | 57 | 38 | |
| Grade | | | |
| 1 | 44 | > 60 | 0.504 |
| 2 | 40 | 51 | |
| 3 | 23 | 35 | |
| Residual disease after primary surgery | | | |
| Optimal | 77 | > 60 | < 0.0001 |
| Suboptimal | 29 | 19 | |
| PRAB LI | | | |
| Negative | 28 | 40 | 0.0207 |
| Positive | 79 | > 60 | |
| PRA LI | | | |
| Negative | 69 | > 60 | 0.24 |
| Positive | 38 | > 60 | |
| PRB LI | | | |
| Negative | 30 | 37 | 0.0117 |
| Positive | 77 | > 60 | |
| ER-αLI | | | |
| Negative | 51 | 52 | 0.0374 |
| Positive | 56 | > 60 | |

[a]Significant difference was observed for endometrioid vs mucinous adenocarcinoma; positive immunoreactivity was defined as LI > 10

**Table 4** Multivariate analysis of survival time using Cox's proportional hazards model

| Covariate | Relative risk | 95% CI[a] | *P* |
|---|---|---|---|
| Performance status | | | |
| 0–1 | 1.000 | | |
| 2–4 | 2.907 | 1.242–6.803 | 0.0138 |
| Histology | | | |
| Serous | 1.000 | | |
| Endometrioid | 2.162 | 0.821–5.698 | 0.119 |
| Clear cell | 1.659 | 0.655–3.788 | 0.177 |
| Mucinous | 0.423 | 0.044–4.082 | 0.457 |
| Stage | | | |
| I–II | 1.000 | | |
| III–IV | 2.565 | 1.252–10.222 | 0.028 |
| Residual disease after primary surgery | | | |
| Optimal | 1.000 | | |
| Suboptimal | 5.150 | 2.000–13.333 | <0.001 |
| PRAB LI | | | |
| Negative | 1.000 | | |
| Positive | 0.210 | 0.039–1.126 | 0.069 |
| PRB LI | | | |
| Negative | 1.000 | | |
| Positive | 0.173 | 0.0333–0.901 | 0.037 |
| ER LI | | | |
| Negative | 1.000 | | |
| Positive | 0.878 | 0.316–2.440 | 0.802 |

[a]CI = confidence interval

2/3; clear cell, 4/5 and 3/5, respectively. The positivities of RT-PCR analysis in each case of ovarian carcinoma were consistent with those of immunohistochemistry.

### DISCUSSION

Human epithelial ovarian carcinoma is believed to be a sex steroid hormone-dependent neoplasm, but the biological significance of PR, especially its role in growth regulation, and its correlation to clinical outcome in patients, has remained in dispute. Several groups of investigators reported no significant difference in the survival of ovarian cancer patients with respect to PR levels (Bizzi et al, 1988; Geisler et al, 1996; Masood et al, 1989), while some other studies have reported that a higher PR status correlated well with increased survival (Inversen et al, 1986; Masood et al, 1989; Slotman et al, 1989; Kommoss et al, 1992; Hempling et al, 1998; Langdon et al, 1998). Review of these reports indicates that the difference in these studies may be due to the heterologous composition of the patient population. For example, several groups of investigators included tumours of low malignant potential and others included only cases with advanced stage of cancer (Masood et al, 1989; Geisler et al, 1996; Hempling et al, 1998). Hempling et al (1998) and Inversen et al (1986) both demonstrated PR as a significant prognostic factor following multivariate analysis, but both examined only advanced (stage III–IV) epithelial ovarian carcinoma. Therefore, in this study we evaluated only epithelial ovarian carcinoma without low malignant potential, in all stages and all major histologic subtypes using a multivariate analysis.

The identification of PR subtypes demonstrates that the analysis of subtypes for PR can provide new insights into the biological roles of PR in human epithelial ovarian carcinoma. In our study, PRB is dominantly expressed in all types or groups of epithelial ovarian carcinoma using both immunohistochemistry and RT-PCR. In addition, PRB, but not PRAB, immunoreactivity turned out to be an independent prognostic factor following multivariate analysis. In all previous studies on PR in ovarian epithelial carcinoma, PR isoforms were not evaluated separately as in PRAB in our present study. Therefore, the discrepancy between the results of previous studies and our present examination may be due to the evaluation of PR subtype in our study, and the analysis of PR isoforms considered important in the evaluation of the survival of patients diagnosed with epithelial ovarian carcinoma. Fujimoto et al (1995) reported the expression of PR isoform mRNAs in ovarian carcinoma using RT-PCR. In their study, six out of ten cases expressed only PRB mRNA. They subsequently concluded that the dominancy of PRB mRNA expression was associated with the advanced clinical stage of ovarian cancer (Fujimoto et al, 1995). Results of their study, especially the dominance of PRB, but not PRA mRNA, were consistent with those of our present study (Fujimoto et al, 1995). Therefore, the dominant expression of PRB is considered to be a characteristic feature of ovarian cancer, in contrast to breast and endometrial carcinoma, in which PRA is the dominant subtype of PR (Kumar et al, 1998; Graham et al, 1996).

The biological role of progesterone in epithelial ovarian carcinoma is unknown, but progesterone is, in general, considered to function antagonistically to oestrogen-mediated cell proliferation (Clarke and Sutherland, 1990). PRA has been reported to be a transcriptional inhibitor of ER (Simpson et al, 1998), and PRB has the ability to up-regulate the transcription of genes required for cell differentiation (Kumar et al, 1998). The transcriptional activation of PRB is more marked than PRA (Kastner et al, 1990), which

*© 2000 Cancer Research Campaign*

suggests that the inhibitory effects on cell proliferation by progesterone is, at least, initially mediated through PRB.

Friedlander et al (1989) described a significantly lower S-phase fraction among PR-positive tumours compared with PR-negative tumours. Additionally, they demonstrated that a significantly greater proportion of diploid tumors were PR-positive than were aneuploid tumours (Friedlander et al, 1989). Both tumour ploidy and proliferative activity, determined by S-phase fraction, have been demonstrated to be significant determinants in the survival of patients with epithelial ovarian cancer (Erhardt et al, 1984; Volm et al, 1985). Therefore, ovarian epithelial carcinoma cases associated with functional PR, especially PRB, and sufficient in situ availability of progesterone are considered to be associated with better clinical outcome. This may be due to the inhibitory actions of progesterone on tumour cell proliferation, but this hypothesis awaits further investigation for clarification. Kumar et al (1998) examined the expression of PRA and PRB in endometrial carcinoma and reported that selective down-regulation of PRB may represent an insufficient response to progestin therapy in patients with poorly differentiated endometrial carcinoma (Kumar et al, 1998). Similar results were also reported in breast carcinoma, i.e. tumours containing primarily PRA related to poor response to endocrine agents (Geisler et al, 1996). These studies have demonstrated that the dominant expression of PRB suggests a good response for progestin in breast and endometrial cancer patients, although the clinical usefulness of sex steroid hormones in the treatment of ovarian cancer has yet to be determined (Ahlgren et al, 1993).

There was, in general, a good correlation between ER-α and PR in epithelial ovarian carcinoma, suggesting that the regulation of PR, especially of PRB, may be under oestrogen control in ovarian epithelial carcinoma, consistent with the results of previous in vitro studies (Kastner et al, 1990; Savouret et al, 1990). Both PRA and PRB mRNAs have been demonstrated to be increased by oestrogen, but between these two isoforms, preferential up-regulation of PRB by oestrogen has been reported in the T47D human breast carcinoma cell line (Graham et al, 1995), and in human endometrial tissue (Mangal et al, 1997). However, it has also been reported that the stimulatory effects of oestrogen on PRA protein levels were greater than PRB in chicken oviduct (Syvala et al, 1997), suggesting that oestrogen stimulation of PRA and PRB is likely to be cell-, tissue-, and species-specific. In addition, mechanisms other than those under ER control can influence PR expression, as was demonstrated in PR-positive, ER-negative breast tumours (Horwitz, 1981). PR expression has been demonstrated to be regulated by growth factors (Katzenellenbogen and Norman, 1990), and ER-α knockout mice continue to express a low level of PR mRNA (Shughrue et al, 1997). Therefore, further investigations are required to clarify the possible mechanisms of regulation of PRA and PRB in human epithelial ovarian carcinoma.

### ACKNOWLEDGEMENTS

This work is in part supported by the grant-in-aid for Cancer Research 7–1 from the Ministry of Health and Welfare, Japan, a grant-in-aid for scientific research area on priority area (A-11137301) from the Ministry of Education, Science and Culture, Japan, a grant-in-aid for Scientific Research (B-11470047) from Japan Society for the Promotion of Science and a grant from The Naitou Foundation and Suzuken Memorial Foundation. We appreciate Dr Toru Tase and Dr Hiroo Tateno for their cooperation in retrieving the specimens of ovarian carcinoma tissues at the Miyagi Prefectural Cancer center, Natori, Japan. We also appreciate Ms Keiko Abe and Ms Ayako Kusumi for their technical assistance. We would like to acknowledge the editing of the manuscript by Mr Andrew D Darnel, Department of Pathology, Tohoku University School of Medicine, Sendai, Japan.

### REFERENCES

Ahlgren JD, Ellison NM, Gottlieb RJ, Laluna F, Lokich JJ, Sinclair PR, Ueno W, Wampler GL, Yeung KY, Alt D and Fryer JG (1993) Hormonal palliation of chemoresistant ovarian cancer: three consecutive Phase II trials of the Mid-Atlantic Oncology Program. *J Clin Oncol* **11**: 1957–1968

Benraad THJ, Friberg CG, Koenders AJM and Kullander S (1980) Do estrogen and progesterone receptors (ER and PR) in metastasizing endometrial cancer predict response to estrogen therapy. *Acta Obstet Gynecol Scand* **59**: 155–159

Bizzi A, Codegoni AM and Landoni F (1988) Steroid receptors in epithelial ovarian cancer: Relation to clinical parameters and survival. *Cancer Res* **48**: 6222–6226

Bloom ND, Tobin EH, Schreiberman B and Degenshein GA (1980) The role of progesterone receptors in the treatment of breast cancer. *Cancer Res* **45**: 2992–2997

Clarke CL and Sutherland RL (1990) Progestin regulation of cellular proliferation. *Endocr Rev* **11**: 266–301

Clarke CL, Zaino PD, Feil PD, Miller JV, Steck ME, Ohlsson-Wilhelm BM and Satyaswaroop PG (1987) Monoclonal antibodies to human progesterone receptor: Characterization by biochemical and immunohistochemical techniques. *Endocrinology* **121**: 1123–1132

Critchley HOD, Wang H, Kelly RW, Gebbie AE and Glasier AF (1998) Progestin receptor isoforms and prostaglandin dehydrogenase in the endometrium of women using a levonorgestrel-releasing intrauterine system. *Hum Reprod* **13**: 1210–1217

Del Campo JM, Felip E, Rubio D, Vidal R, Bermejo B, Colomer R and Zanon V (1994) Long-term survival in advanced ovarian cancer after cytoreduction and chemotherapy treatment. *Gynecol Oncol* **53**: 27–32

Ehrlich CE, Young PLM and Cleary RE (1981) Cytoplasmic progesterone and estradiol receptors in normal, hyperplastic and carcinomatous endometria: Therapeutic implications. *Am J Obstet Gynecol* **141**: 539–546

Erhardt K, Aver G, Bjorkholm E, Forsslund G, Moberger B, Silfversward C, Wicksell G and Zetterberg A (1984) Prognostic significance of DNA content in serous ovarian tumors. *Cancer Res* **44**: 2198–2202

Evans RM (1988) The steroid and thyroid hormone receptor superfamily. *Science* **240**: 889–895

Friedlander M, Quinn MA, Fontune D, Foo MS, Toppila M, Hudson CN and Russell P (1989) The relationship of steroid receptor expression to nuclear DNA distribution and clinicopathologic characteristics in epithelial ovarian tumors. *Gynecol Oncol* **32**: 184–190

Fujimoto J, Ichigo S, Hori M, Nishigaki M and Tamaya T (1995) Expression of progesterone receptor form A and B mRNAs in gynecologic malignant tumors. *Tumor Biol* **16**: 254–260

Fujimoto J, Hirose R, Ichigo S, Sakaguchi H, Li Y and Tamaya T (1998) Expression of progesterone receptor form A and B mRNAs in uterine leiomyoma. *Tumor Biol* **19**: 126–131

Geisler JP, Wiemann MC, Miller GA and Geisler HE (1996) Estrogen and progesterone receptor status as prognostic indicators in patients with optimally cytoreduced stage IIIc serous cystadenocarcinoma of the ovary. *Gynecol Oncol* **60**: 424–427

Giangrande PH, Pollio G and McDonnell DP (1997) Mapping and characterization of the functional domains responsible for the differential activity of the A and B isoforms of the human progesterone receptor. *J Biol Chem* **272**: 32889–32900

Graham JD, Roman SD, McGowan EM, Sutherland RL and Clarke CL (1995) Preferential stimulation of human progesterone receptor B expression by estrogen in T47D human breast cancer cell lines. *J Biol Chem* **270**: 30693–30700

Graham JD, Yeates C, Balleine RL, Harvey SS, Milliken JS, Bilous AM and Clarke CL (1996) Progesterone receptor A and B protein expression in human breast cancer. *J Steroid Biochem Mol Biol* **56**: 93–98

Heintz APM, Hacker NF, Berek JS, Rose TP, Munoz AK and Lagasse LD (1986) Cytoreductive surgery in ovarian carcinoma: Feasibility and morbidity. *Obstet Gynecol* **67**: 783–788

*© 2000 Cancer Research Campaign* 

Hempling RE, Piver MS, Eltabbakh GH and Recio FO (1998) Progesterone receptor status is a significant prognostic variable of progression-free survival in advanced epithelial ovarian cancer. *Am J Clin Oncol* **21**: 447–451

Hirasawa G, Sasano H, Takahashi K, Fukushima K, Suzuki T, Hiwatashi N, Toyota T, Krozowski ZS and Nagura H (1997) Colocalization of 11β-hydroxysteroid dehydrogenase type II and mineralcorticoid receptor in human epithelia. *J Clin Endoclinol Metab* **82**: 3859–3863

Horwitz KB (1981) Is a functional estrogen receptor always required for progesterone receptor induction in breast cancer? *J Steroid Biochem* **15**: 209–217

Horwitz KB and Alexander PS (1983) In situ photolinked nuclear progesterone receptors of human breast cancer cell lines: subunit molecular weights after transformation and translocation. *Endocrinology* **113**: 2195–2201

International Federation of Gynecology and Obstetrics (1987) Changes in definitions of clinical staging for adenocarcinoma of the cervix and ovary. *Am J Obstet Gynecol* **156**: 263–264

Inversen OE, Skaarland E and Utaaker E (1986) Steroid receptor content in human ovarian tumors: Survival of patients with ovarian carcinoma related to steroid receptor content. *Gynecol Oncol* **23**: 65–76

Jeltsch JM, Krozowski Z, Qurin-Stricker C, Gronemeyer H, Simpson RJ, Garnier JM, Krust A, Jacob F and Chambon P (1986) Cloning of the chicken progesterone receptor. *Proc Natl Acad Sci USA* **83**: 5424–5428

Kastner P, Krust A, Turcotte B, Stropp U, Tora L, Gronemeyer H and Chambon P (1990) Two distinct estrogen-regulated promoters generate transcripts encoding the two functionally different human progesterone receptor forms A and B. *EMBO J* **9**: 1603–1614

Katzenellenbogen BS and Norman MJ (1990) Multihormonal regulation of the progesterone receptor in MCF-7 human breast cancer cells: Interrelationships among insulin/insulin-like growth factor-1, serum, and estrogen. *Endocrinology* **126**: 891–898

Kaupilla A (1984) Progestin therapy of endometrial, breast and ovarian carcinoma: A review of clinical observations. *Acta Obstet Gynecol Scand* **63**: 441–450

Kommoss F, Pfisterer J, Thome M, Schafer W, Sauerbrei W and Pfleiderer A (1992) Steroid receptors in ovarian carcinoma: Immunohistochemical determination may lead to new aspects. *Gynecol Oncol* **47**: 317–322

Kraus WL, Weis KE and Katzenellenbogen BS (1995) Inhibitory cross-talk between steroid hormone receptors: differential targeting of estrogen receptor in the repression of its transcriptional activity by agonist- and antagonist-occupied progestin receptors. *Mol Cell Biol* **15**: 1847–1857

Kumar NS, Richer J, Owen G, Litman E, Horwitz KB and Leslie KK (1998) Selective down-regulation of progesterone receptor isoforms B in poorly differentiated human endometrial cancer cells: Implications for unopposed estrogen action. *Cancer Res* **58**: 1860–1865

Langdon SP, Gabra H, Bartlett JMS, Rabiaz GJ, Hawkins RA,f Tesdale AL, Ritchie AA, Miller WR and Smyth JF (1998) Functionality of the progesterone receptor in ovarian cancer and its regulation by estrogen. *Clin Cancer Res* **4**: 2245–2251

Mangal RK, Wiehle RD, Poindexter AN and Weigel NL (1997) Differential expression of uterine progesterone receptor forms A and B during the menstrual cycle. *J Steroid Biochem Molec Biol* **63**: 195–202

Masood S, Heitmann J, Nuss RC and Benrubi GI (1989) Clinical correlation of hormone receptor status in epithelial ovarian cancer. *Gynecol Oncol* **34**: 57–60

McGuire WL (1978) Steroid receptors in human breast cancer. *Cancer Res* **38**: 4289–4291

Mote PA, Balleine RL, McGowan EM and Clarke CL (1999) Colocalization of progesterone receptors A and B by dual immunofluorescent histochemistry in human endometrium during the menstrual cycle. *J Clin Endocrinol Metabol* **84**: 2963–2971

Nakashima N, Nagasaka T, Fukata S, Oiwa N, Nara Y, Fukatsu T and Takeuchi J (1990) Study of ovarian tumors treated at Nagoya University Hospital, 1965–1988. *Gynecol Oncol* **37**: 103–111

Omura GA, Brady MF, Homesley HD, Yordan E, Major FJ, Buchsbaum HJ and Park RC (1991) Long-term follow-up and prognostic factor analysis in advanced ovarian carcinoma: The gynecologic oncology group experience. *J Clin Oncol* **9**: 1138–1150

Osborne CK, Yochmowitz MG, Knight WA and McGuire WL (1980) The value of estrogen and progesterone receptors in the treatment of breast cancer. *Cancer* **46**: 2884–2888

Pettersson F (1994) Annual report on the results of treatment in gynecological cancer. *Int J Gynaecol Obstet* **22**: 83–102

Piver MS, Lele SB, Marchetti DL, Baker TR, Tsukada Y and Emrich LJ (1988) The impact of aggressive debulking surgery and cisplatin-based chemotherapy on progression-free survival in stage III and IV ovarian carcinoma. *J Clin Oncol* **6**: 983–989

Rao BR and Slotman BJ (1991) Endocrine factors in common epithelial ovarian cancer. *Endocrine Rev* **12**: 14–26

Redman JR, Petrini GR, Saigo PE, Geller NL and Hakes TB (1986) Prognostic factors in advanced ovarian carcinoma. *J Clin Oncol* **4**: 515–523

Sambrook J, Fritsch EF and Maniatis T (year?) *Molecular cloning, a laboratory manual*, 2nd edn. *Extraction, purification and analysis of messenger RNA from eukaryotic cells, Vol 1*, pp 7.1–7.87. Spring Harbor Laboratory Press: Plainview, New York

Sasano H, Frost AR, Saitoh R, Harada N, Poutanen M, Vihko R, Bulin SE, Silverberg SG and Nagura H (1996) Aromatase and 17 β-hydroxysteroid dehydrogenase type 1 in human breast carcinoma. *J Clin Endoclinol Metab* **81**: 4042–4046

Savouret JF, Misrahi M and Milgrom E (1990) Molecular action of progesterone. *Int J Biochem* **22**: 579–594

Sevelda P, Denison U, Schemper M, Spona J, Vavra N and Salzer H (1990) Oestrogen and progesterone receptor content as a prognostic factor in advanced epithelial ovarian carcinoma. *Br J Obstet Gynecol* **97**: 706–712

Shughrue PJ, Lubahn DB, Negro-Vilar A, Korach KS and Merchenthaler I (1997) Responses in the brain of estrogen receptor α-disrupted mice. *Proc Natl Acad Sci USA* **94**: 11008–11012

Simpson BJB, Langdon SP, Rabiasz GJ, Macleod KG, Hirst GL, Bartlett JMS, Crew AJ, Hawkins RA, Macineira-Perez PP, Smyth JF and Miller WR (1998) Estrogen regulation of transforming growth factor-α in ovarian cancer. *J Steroid Biochem Mol Biol* **64**: 137–145

Slotman BJ, Kuhnel R, Rao BR, Dijkhuizen GH, deGraaf J and Stolk JG (1989) Importance of steroid receptors and aromatase activity in the prognosis of ovarian cancer: High tumor progesterone receptor levels correlate with longer survival. *Gynecol Oncol* **33**: 76–81

Syvala H, Vienonen A, Ylikomi T, Blauer M, Zhuang YH and Tuohimaa P (1997) Expression of the chick progesterone receptor forms A and B is differentially regulated by estrogen in vivo. *Biochem Biophys Res Commun* **231**: 573–576

Vegeto E, Shahbaz MM, Wen DX, Goldman ME, O'Malley BW and McDonell DP (1993) Human progesterone receptor A form is a cell- and promoter-specific repress or of human progesterone receptor B function. *Mol Endocrinol* **7**: 1244–1255

Viville B, Charnock-Jones DS, Sharkey AM, Wetzka B and Smith SK (1997) Distribution of the A and B forms of the progesterone receptor messenger ribonucleic acid and protein in uterine leiomyomata and adjacent myometrium. *Hum Reprod* **12**: 815–822

Volm M, Bruggeman A, Gunther M, Kleine W, Pfleiderer A and Vogt-Schaden M (1985) Prognostic significance of ploidy and proliferation in ovarian carcinoma. *Cancer Res* **45**: 5180–5185

Wang H, Critchley HOD, Kelly RW, Shen D and Baird DT (1998) Progesterone receptor subtype B is differentially regulated in human endometrial stroma. *Mol Hum Reprod* **4**: 407–412

Wen DX, Xu YF, Mais DE, Godman ME and McDonnell DP (1994) The A and B isoform of the human progesterone receptor operate through distinct signaling pathways within target cells. *Mol Cell Biol* **14**: 8356–8364

World Health Organization (1979) *Handbook for reporting results of cancer treatment. WHO publication no. 48*. World Health Organization: Geneva

Young RC, Chabner BA, Hubbard SP, Fischer RI, Bender RA, Anderson T, Simon RM, Canellos GP and De Vita VT (1978) Advanced ovarian adenocarcinoma: A prospective clinical trial of melphalan (L-PAM) versus combination chemotherapy. *New Engl J Med* **299**: 1261–1266

*© 2000 Cancer Research Campaign*

## References

[PDF_00561] Ahlgren J. D., Ellison N. M., Gottlieb R. J., Laluna F., Lokich J. J., Sinclair P. R., Ueno W., Wampler G. L., Yeung K. Y., Alt D. (1993). Hormonal palliation of chemoresistant ovarian cancer: three consecutive phase II trials of the Mid-Atlantic Oncology Program.. J Clin Oncol.

[PDF_00565] Benraad T. J., Friberg L. G., Koenders A. J., Kullander S. (1980). Do estrogen and progesterone receptors (E2R and PR) in metastasizing endometrial cancers predict the response to gestagen therapy?. Acta Obstet Gynecol Scand.

[PDF_00568] Bizzi A., Codegoni A. M., Landoni F., Marelli G., Marsoni S., Spina A. M., Torri W., Mangioni C. (1988). Steroid receptors in epithelial ovarian carcinoma: relation to clinical parameters and survival.. Cancer Res.

[PDF_00571] Bloom N. D., Tobin E. H., Schreibman B., Degenshein G. A. (1980). The role of progesterone receptors in the management of advanced breast cancer.. Cancer.

[PDF_00574] Clarke C. L., Sutherland R. L. (1990). Progestin regulation of cellular proliferation.. Endocr Rev.

[PDF_00576] Clarke C. L., Zaino R. J., Feil P. D., Miller J. V., Steck M. E., Ohlsson-Wilhelm B. M., Satyaswaroop P. G. (1987). Monoclonal antibodies to human progesterone receptor: characterization by biochemical and immunohistochemical techniques.. Endocrinology.

[PDF_00580] Critchley H. O., Wang H., Kelly R. W., Gebbie A. E., Glasier A. F. (1998). Progestin receptor isoforms and prostaglandin dehydrogenase in the endometrium of women using a levonorgestrel-releasing intrauterine system.. Hum Reprod.

[PDF_00584] Del Campo J. M., Felip E., Rubio D., Vidal R., Bermejo B., Colomer R., Zanon V. (1994). Long-term survival in advanced ovarian cancer after cytoreduction and chemotherapy treatment.. Gynecol Oncol.

[PDF_00587] Ehrlich C. E., Young P. C., Cleary R. E. (1981). Cytoplasmic progesterone and estradiol receptors in normal, hyperplastic, and carcinomatous endometria: therapeutic implications.. Am J Obstet Gynecol.

[PDF_00590] Erhardt K., Auer G., Björkholm E., Forsslund G., Moberger B., Sifverswärd C., Wicksell G., Zetterberg A. (1984). Prognostic significance of nuclear DNA content in serous ovarian tumors.. Cancer Res.

[PDF_00593] Evans R. M. (1988). The steroid and thyroid hormone receptor superfamily.. Science.

[PDF_00595] Friedlander M. L., Quinn M. A., Fortune D., Foo M. S., Toppila M., Hudson C. N., Russell P. (1989). The relationship of steroid receptor expression to nuclear DNA distribution and clinicopathological characteristics in epithelial ovarian tumors.. Gynecol Oncol.

[PDF_00602] Fujimoto J., Hirose R., Ichigo S., Sakaguchi H., Li Y., Tamaya T. (1998). Expression of progesterone receptor form A and B mRNAs in uterine leiomyoma.. Tumour Biol.

[PDF_00599] Fujimoto J., Ichigo S., Hori M., Nishigaki M., Tamaya T. (1995). Expression of progesterone receptor form A and B mRNAs in gynecologic malignant tumors.. Tumour Biol.

[PDF_00605] Geisler J. P., Wiemann M. C., Miller G. A., Geisler H. E. (1996). Estrogen and progesterone receptor status as prognostic indicators in patients with optimally cytoreduced stage IIIc serous cystadenocarcinoma of the ovary.. Gynecol Oncol.

[PDF_00609] Giangrande P. H., Pollio G., McDonnell D. P. (1997). Mapping and characterization of the functional domains responsible for the differential activity of the A and B isoforms of the human progesterone receptor.. J Biol Chem.

[PDF_00613] Graham J. D., Roman S. D., McGowan E., Sutherland R. L., Clarke C. L. (1995). Preferential stimulation of human progesterone receptor B expression by estrogen in T-47D human breast cancer cells.. J Biol Chem.

[PDF_00617] Graham J. D., Yeates C., Balleine R. L., Harvey S. S., Milliken J. S., Bilous A. M., Clarke C. L. (1996). Progesterone receptor A and B protein expression in human breast cancer.. J Steroid Biochem Mol Biol.

[PDF_00620] Heintz A. P., Hacker N. F., Berek J. S., Rose T. P., Munoz A. K., Lagasse L. D. (1986). Cytoreductive surgery in ovarian carcinoma: feasibility and morbidity.. Obstet Gynecol.

[PDF_00625] Hempling R. E., Piver M. S., Eltabbakh G. H., Recio F. O. (1998). Progesterone receptor status is a significant prognostic variable of progression-free survival in advanced epithelial ovarian cancer.. Am J Clin Oncol.

[PDF_00628] Hirasawa G., Sasano H., Takahashi K., Fukushima K., Suzuki T., Hiwatashi N., Toyota T., Krozowski Z. S., Nagura H. (1997). Colocalization of 11 beta-hydroxysteroid dehydrogenase type II and mineralocorticoid receptor in human epithelia.. J Clin Endocrinol Metab.

[PDF_00635] Horwitz K. B., Alexander P. S. (1983). In situ photolinked nuclear progesterone receptors of human breast cancer cells: subunit molecular weights after transformation and translocation.. Endocrinology.

[PDF_00632] Horwitz K. B. (1981). Is a functional estrogen receptor always required for progesterone receptor induction in breast cancer?. J Steroid Biochem.

[PDF_00641] Iversen O. E., Skaarland E., Utaaker E. (1986). Steroid receptor content in human ovarian tumors: survival of patients with ovarian carcinoma related to steroid receptor content.. Gynecol Oncol.

[PDF_00644] Jeltsch J. M., Krozowski Z., Quirin-Stricker C., Gronemeyer H., Simpson R. J., Garnier J. M., Krust A., Jacob F., Chambon P. (1986). Cloning of the chicken progesterone receptor.. Proc Natl Acad Sci U S A.

[PDF_00647] Kastner P., Krust A., Turcotte B., Stropp U., Tora L., Gronemeyer H., Chambon P. (1990). Two distinct estrogen-regulated promoters generate transcripts encoding the two functionally different human progesterone receptor forms A and B.. EMBO J.

[PDF_00651] Katzenellenbogen B. S., Norman M. J. (1990). Multihormonal regulation of the progesterone receptor in MCF-7 human breast cancer cells: interrelationships among insulin/insulin-like growth factor-I, serum, and estrogen.. Endocrinology.

[PDF_00655] Kauppila A. (1984). Progestin therapy of endometrial, breast and ovarian carcinoma. A review of clinical observations.. Acta Obstet Gynecol Scand.

[PDF_00657] Kommoss F., Pfisterer J., Thome M., Schäfer W., Sauerbrei W., Pfleiderer A. (1992). Steroid receptors in ovarian carcinoma: immunohistochemical determination may lead to new aspects.. Gynecol Oncol.

[PDF_00660] Kraus W. L., Weis K. E., Katzenellenbogen B. S. (1995). Inhibitory cross-talk between steroid hormone receptors: differential targeting of estrogen receptor in the repression of its transcriptional activity by agonist- and antagonist-occupied progestin receptors.. Mol Cell Biol.

[PDF_00664] Kumar N. S., Richer J., Owen G., Litman E., Horwitz K. B., Leslie K. K. (1998). Selective down-regulation of progesterone receptor isoform B in poorly differentiated human endometrial cancer cells: implications for unopposed estrogen action.. Cancer Res.

[PDF_00668] Langdon S. P., Gabra H., Bartlett J. M., Rabiaz G. J., Hawkins R. A., Tesdale A. L., Ritchie A. A., Miller W. R., Smyth J. F. (1998). Functionality of the progesterone receptor in ovarian cancer and its regulation by estrogen.. Clin Cancer Res.

[PDF_00672] Mangal R. K., Wiehle R. D., Poindexter A. N., Weigel N. L. (1997). Differential expression of uterine progesterone receptor forms A and B during the menstrual cycle.. J Steroid Biochem Mol Biol.

[PDF_00675] Masood S., Heitmann J., Nuss R. C., Benrubi G. I. (1989). Clinical correlation of hormone receptor status in epithelial ovarian cancer.. Gynecol Oncol.

[PDF_00677] McGuire W. L. (1978). Steroid receptors in human breast cancer.. Cancer Res.

[PDF_00679] Mote P. A., Balleine R. L., McGowan E. M., Clarke C. L. (1999). Colocalization of progesterone receptors A and B by dual immunofluorescent histochemistry in human endometrium during the menstrual cycle.. J Clin Endocrinol Metab.

[PDF_00683] Nakashima N., Nagasaka T., Fukata S., Oiwa N., Nara Y., Fukatsu T., Takeuchi J. (1990). Study of ovarian tumors treated at Nagoya University Hospital, 1965-1988.. Gynecol Oncol.

[PDF_00686] Omura G. A., Brady M. F., Homesley H. D., Yordan E., Major F. J., Buchsbaum H. J., Park R. C. (1991). Long-term follow-up and prognostic factor analysis in advanced ovarian carcinoma: the Gynecologic Oncology Group experience.. J Clin Oncol.

[PDF_00690] Osborne C. K., Yochmowitz M. G., Knight W. A., McGuire W. L. (1980). The value of estrogen and progesterone receptors in the treatment of breast cancer.. Cancer.

[PDF_00695] Piver M. S., Lele S. B., Marchetti D. L., Baker T. R., Tsukada Y., Emrich L. J. (1988). The impact of aggressive debulking surgery and cisplatin-based chemotherapy on progression-free survival in stage III and IV ovarian carcinoma.. J Clin Oncol.

[PDF_00699] Rao B. R., Slotman B. J. (1991). Endocrine factors in common epithelial ovarian cancer.. Endocr Rev.

[PDF_00701] Redman J. R., Petroni G. R., Saigo P. E., Geller N. L., Hakes T. B. (1986). Prognostic factors in advanced ovarian carcinoma.. J Clin Oncol.

[PDF_00707] Sasano H., Frost A. R., Saitoh R., Harada N., Poutanen M., Vihko R., Bulun S. E., Silverberg S. G., Nagura H. (1996). Aromatase and 17 beta-hydroxysteroid dehydrogenase type 1 in human breast carcinoma.. J Clin Endocrinol Metab.

[PDF_00711] Savouret J. F., Misrahi M., Milgrom E. (1990). Molecular action of progesterone.. Int J Biochem.

[PDF_00713] Sevelda P., Denison U., Schemper M., Spona J., Vavra N., Salzer H. (1990). Oestrogen and progesterone receptor content as a prognostic factor in advanced epithelial ovarian carcinoma.. Br J Obstet Gynaecol.

[PDF_00716] Shughrue P. J., Lubahn D. B., Negro-Vilar A., Korach K. S., Merchenthaler I. (1997). Responses in the brain of estrogen receptor alpha-disrupted mice.. Proc Natl Acad Sci U S A.

[PDF_00719] Simpson B. J., Langdon S. P., Rabiasz G. J., Macleod K. G., Hirst G. L., Bartlett J. M., Crew A. J., Hawkins R. A., Macineira-Perez P. P., Smyth J. F. (1998). Estrogen regulation of transforming growth factor-alpha in ovarian cancer.. J Steroid Biochem Mol Biol.

[PDF_00723] Slotman B. J., Kühnel R., Rao B. R., Dijkhuizen G. H., de Graaff J., Stolk J. G. (1989). Importance of steroid receptors and aromatase activity in the prognosis of ovarian cancer: high tumor progesterone receptor levels correlate with longer survival.. Gynecol Oncol.

[PDF_00727] Syvälä H., Vienonen A., Ylikomi T., Bläuer M., Zhuang Y. H., Tuohimaa P. (1997). Expression of the chicken progesterone receptor forms A and B is differentially regulated by estrogen in vivo.. Biochem Biophys Res Commun.

[PDF_00730] Vegeto E., Shahbaz M. M., Wen D. X., Goldman M. E., O'Malley B. W., McDonnell D. P. (1993). Human progesterone receptor A form is a cell- and promoter-specific repressor of human progesterone receptor B function.. Mol Endocrinol.

[PDF_00734] Viville B., Charnock-Jones D. S., Sharkey A. M., Wetzka B., Smith S. K. (1997). Distribution of the A and B forms of the progesterone receptor messenger ribonucleic acid and protein in uterine leiomyomata and adjacent myometrium.. Hum Reprod.

[PDF_00738] Volm M., Brüggemann A., Günther M., Kleine W., Pfleiderer A., Vogt-Schaden M. (1985). Prognostic relevance of ploidy, proliferation, and resistance-predictive tests in ovarian carcinoma.. Cancer Res.

[PDF_00741] Wang H., Critchley H. O., Kelly R. W., Shen D., Baird D. T. (1998). Progesterone receptor subtype B is differentially regulated in human endometrial stroma.. Mol Hum Reprod.

[PDF_00744] Wen D. X., Xu Y. F., Mais D. E., Goldman M. E., McDonnell D. P. (1994). The A and B isoforms of the human progesterone receptor operate through distinct signaling pathways within target cells.. Mol Cell Biol.

[PDF_00749] Young R. C., Chabner B. A., Hubbard S. P., Fisher R. I., Bender R. A., Anderson T., Simon R. M., Canellos G. P., DeVita V. T. (1978). Advanced ovarian adenocarcinoma. A prospective clinical trial of melphalan (L-PAM) versus combination chemotherapy.. N Engl J Med.

